# Zinc–Tin Oxide Film as an Earth-Abundant Material and Its Versatile Applications to Electronic and Energy Materials

**DOI:** 10.3390/membranes12050485

**Published:** 2022-04-29

**Authors:** Juhyung Seo, Hocheon Yoo

**Affiliations:** Department of Electronic Engineering, Gachon University, Seongnam 13120, Korea; qaz4317@gachon.ac.kr

**Keywords:** oxide semiconductor film, zinc–tin oxide, thin-film transistors, flexible electronics, memory, solar cell, sensor application

## Abstract

Zinc–Tin Oxide (ZTO) films potentially offer desirable properties for next-generation devices and are considered promising candidates due to the following merits: (I) zinc and tin are abundant on Earth, with estimated reserves of approximately 250 million tons and 4.3 billion tons, respectively, (II) zinc and tin are harmless to the human body, and (III) large-area manufacturing with various synthesis processes is available. Considering the advantages and promises of these ZTO films, this review provides a timely overview of the progress and efforts in developing ZTO-based electronic and energy devices. This review revisits the ZTO films used for various device applications, including thin-film transistors, memory devices, solar cells, and sensors, focusing on their strong and weak points. This paper also discusses the opportunities and challenges for using ZTO films in further practical electronic and energy device applications.

## 1. Introduction

Zinc–tin oxide (ZTO) is a wide bandgap semiconductor (Eg = ~3.6 eV) [1,2,3,4] that has been studied as a potential material for electronic and energy devices, such as thin-film transistors [5,6,7,8], versatile sensors [9,10], and solar cells [11,12,13]. The ZTO film has promising properties, including transparency [14,15,16], flexibility [14,15], and semiconducting ability [6], with high electron charge transport behavior that offers desirable electronic and energy materials. Another merit of the ZTO films is that they have high process compatibility. ZTO films can be synthesized using solution- and vacuum-based processes [17]. Various synthesis approaches have been developed for ZTO films, owing to their compatibility, including sputtering [18,19,20,21], atomic layer deposition (ALD) [3,8,22,23], chemical vapor deposition (CVD) [24,25,26,27], spin-coating [28,29,30,31,32], and printing techniques [6,7,33,34].

The unique advantage of the ZTO films is the abundance of resources. Zinc and tin are abundant on Earth, with estimated reserves of approximately 250 million tons [35] and 4.3 billion tons [35], respectively. These values are a hundred and a thousand-times larger than those of other metal oxide compositions. For example, gallium and indium are by-products from the refining process of other minerals but only have reserves of approximately 1 million tons [36] and 255,000 tons [37], respectively. The low rarity of the ZTO can avoid the issues of process-cost increase accompanying the resource scarcity problem due to material depletion.

ZTO is expected to have a range of applications in the future. In this context, this review article provides an overview of ZTO-based electronic and energy applications. Section 2.1 summarizes synthesis methods classified by the solution process and the recent advances in patterning processability. Section 2.2 reviews the development of thin-film transistors using ZTO films, with respect to their charge transport properties, depending on device structure and fabrication process aspects. Section 2.3 introduces recent efforts in flexible electronics using the ZTO films. Section 2.4 summarizes the floating gate memory, using ZTO as the active layer and neuromorphic application using ZTO-based resistive memory, memristors. The progress in solar cells using ZTO films is reviewed in Section 2.5, focusing on their operation mechanism. Section 2.6 summarizes newly developed sensing applications with ZTO films as the active layer. Section 3 discusses the challenges and opportunities in the versatile use of ZTO in electronic and energy devices.

## 2. ZTO Film and Applications

### 2.1. Manufacturing Process of ZTO Thin Film

**Figure 1:**
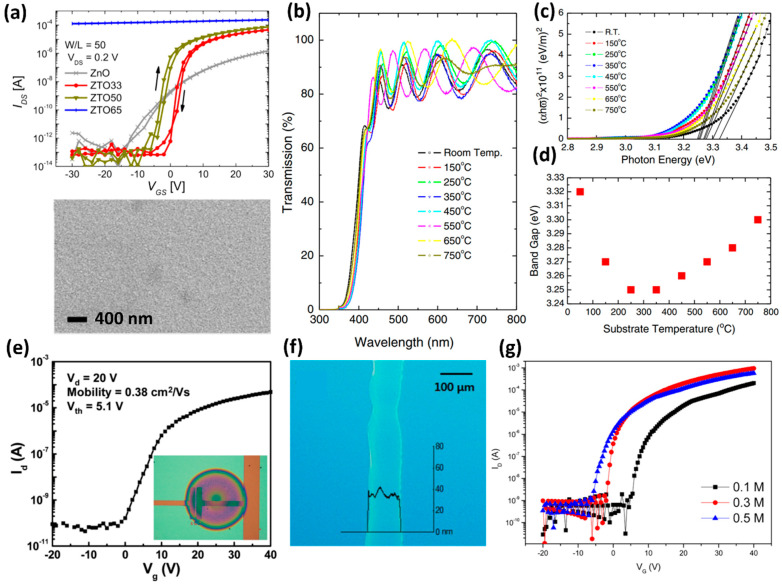
(**a**) Transfer characteristics of the ZTO TFT depending on the adjusted tin ratio. The TFT was measured as V_DS_ = 0.2 V, and plan view scanning electron microscopy (SEM) image of ZTO films (adapted from [40] with permission from John Wiley and Sons). (**b**) Transmittance of the fabricated ZTO film, and (**c**) relationship between ${\left(ahv\right)}^{2}$ and hv depending on the substrate temperature. (**d**) The optical band gap of ZTO film according to the substrate temperature (adapted from [2] with permission from Elsevier). (**e**) Transfer characteristics of the inkjet-printed transistors (adapted from [48] with permission from American Chemical Society). (**f**) Optical microscopy image of the patterned ZTO by EHD-jet. (**g**) Transfer characteristics of the EHD-jet printed ZTO TFT with different mole ratios (adapted from [49] with permission from the American Chemical Society).

Among the methods of synthesizing oxide semiconductors, sputtering, which has high compatibility with conventional silicon processes, allows easy patterning using shadow masks and photolithography. Specifically, in ZTO films, the electrical properties of semiconductors are controlled easily, according to the zinc and tin composition ratio. The characteristics of the ZTO film can be customized according to the applications by adjusting the composition ratio of the target in the sputtering process. Therefore, the synthesis of the ZTO film using sputtering has been studied extensively [38,39]. In 2017, Niang et al. synthesized a ZTO film on a Si/SiO_2_ substrate by sputtering and analyzed the electrical characteristics of the ZTO film according to the ratio of tin [40]. In ZTO oxide semiconductors, tin is an essential element that determines the electrical conductivity of the semiconductor. In ZTO films with a zero-tin ratio (ZnO), the film has a relatively poor carrier mobility of 0.7 cm^2^/V∙s and an on–off ratio of 10^6^. The carrier mobility and on–off ratio increased gradually with the increasing tin ratio. At a tin ratio of 50%, the ZTO film showed the highest carrier mobility (20.2 cm^2^/V∙s) and the largest on–off ratio (5 × 10^8^). On the other hand, the off state no longer existed at tin ratios above 65% because channel depletion could not be achieved (Figure 1a).

In 2013, Lee et al. analyzed the structural, optical, and electrical properties of ZTO films, according to the substrate temperature in the sputtering process [2]. The film was deposited by sputtering on heated glass, at various substrate temperature variations. The ZTO film showed various properties, depending on the substrate temperature. In particular, the fabricated film transmits the most visible light wavelengths under all conditions (Figure 1b). XRD showed that the film had an amorphous structure on a substrate below 350 °C, and as the temperature increased, the film crystallized between 450–550 °C and transformed into ZnSnO_3_. At temperatures above 650 °C, the film crystallized and transformed into Zn_2_SnO_4_. Hence, the bandgap of ZTO films depends on the substrate temperature. As shown in Figure 1c,d, the bandgap of the film changed according to the change in substrate temperature. This means that it will be a key material candidate for bandgap engineering in the near future. Nevertheless, the fabrication of ZTO films using sputtering is a complex manufacturing process, involving a high vacuum. Hence, inexpensive and straightforward solution-based processes for ZTO synthesis have been investigated [41,42,43]. In the case of ZTO, however, patterning is complicated with solution process-based film synthesis, and ZTO crystallizes at approximately 500 °C. Furthermore, ZTO thin films have a grim future because the high process temperature is incompatible with vertical stacking technology or flexible electronics.

To avoid the high-temperature annealing process, in 2012, Hwang et al. applied a UV-light annealing process to achieve the low-temperature synthesis of ZTO thin films [44]. The spin-coated ZTO precursor showed the highest absorption at approximately 280 nm, which means that the 280 nm UV light is an optimal condition for light annealing. The UV-light-annealed ZTO film was improved by generating oxygen vacancies with post-vacuum annealing at 250 °C. Hence, the ZTO films synthesized at low temperatures will be compatible with flexible electronics in the future. On the other hand, UV light at 280 nm can damage the underlying device in monolithic integrations, such as vertical stacking. Therefore, in 2017, Salgueiro et al. used combustion chemistry to synthesize a highly crystalline ZTO film with low-temperature annealing, using localized heating [45]. According to combustion chemistry, some organic solvents can operate as a solvent and a fuel in a combustion reaction. High-performance ZTO films can be synthesized at low processing temperatures because the heat generated in the combustion reaction is sufficient to anneal the localized film. ZTO can have benefits in some areas, such as flexible electronics and vertical stacking, owing to the low process temperature. On the other hand, the absence of a patterning method for solution-process-based semiconductors was highlighted as a problem, delaying the commercialization of many semiconductor materials, including ZTO. Various techniques have been developed for patterning in solution processes to solve this problem. For example, there are patterning methods using localized UV irradiation, surface energy differences, or inkjet printing [46] and spray-printing methods [47]. These techniques are attractive techniques for pattern fabrication and precise material transfer because they bypass the need for conventional photolithography and vacuum deposition methods. In 2008, Kim et al. patterned the ZTO channel layer by inkjet printing the ZTO precursor solution between the electrodes [48]. In addition, the performance was analyzed under the condition of the surface (pre-coated IPA, pre-coated hexamethyldisilazane (HMDS), and substrate temperature) to be patterned. All the fabricated thin-film transistors (TFTs) showed an ideal on–off ratio of approximately 10^6^. Therefore, the patterning technique using inkjet printing can be considered an effective patterning candidate in the solution process (Figure 1e). In 2014, Lee et al. obtained patterned ZTO films using electrohydrodynamic-jet printing [49]. This technique is a sophisticated inkjet-printing process that controls the solution with a capillary needle and a high voltage. A tailored jetting profile for the ZTO precursor was investigated as an ideal ZTO film. The precursor solution showed a cone-jet-type nozzle with the dynamic equilibrium of gravity and electric force, at a high voltage of 2.5 kV, which is the most appropriate nozzle shape for patterning. As shown in Figure 1f, the ZTO film was patterned as thin as 60 μm using the optimized cone-jet profile. In addition, a high oxygen vacancy was observed by X-ray photoelectron spectroscopy (XPS). Accordingly, the ZTO TFT had a high carrier mobility of 9.82 cm^2^/V∙s and an on–off ratio of 3.68 × 10^6^ (Figure 1g).

Each of the above-mentioned process approaches has its own set of strengths and limitations. The sputtering method requires a high-cost and complex-to-fabricate vacuum process, but it enables one to obtain high uniformity in film quality [2,40]. Meanwhile, the solution-process approaches, such as spin coating, dipping, and bar coating, have the merit of a low-cost process [41,42,43], but these processes suffer from the difficulty of patterning. Different from the conventional solution processes, inkjet or screen printing offers simple-to-patterned deposition of the ZTO without an additional patterning process [46,48,49]. To reduce the processing temperature of the ZTO synthesis, alternative techniques, such as UV annealing [44] and combustion method [45], were reported.

### 2.2. ZTO-Based Thin-Film Transistors

**Figure 2:**
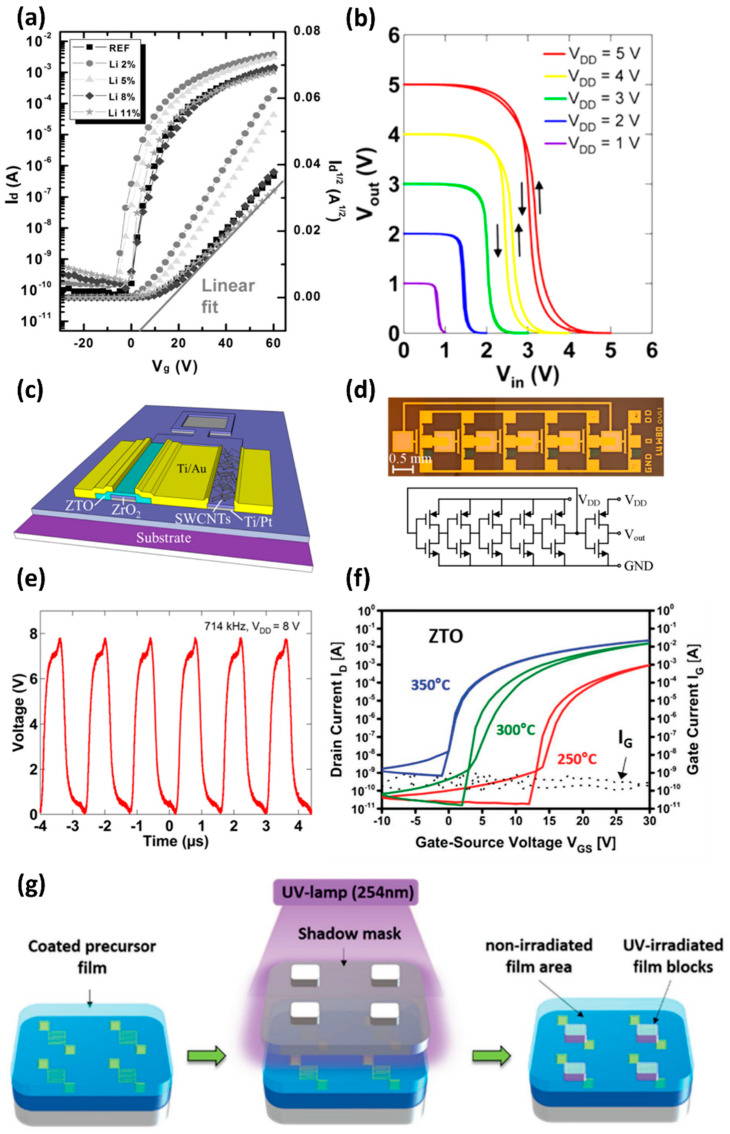
(**a**) Transfer characteristics of the pristine ZTO TFT and Li-doped ZTO TFTs at various Li doping concentrations (adapted from [58] with permission from John Wiley and Sons). (**b**) Characteristic curve of inverter circuit manufactured by combining ZTO TFT and SWCNT TFT. (**c**) The structure of an inkjet-printed ZTO and SWCNT-based complementary inverter. (**d**) Circuit optical image and diagram of a five-stage ROSC. The last inverter is a buffer stage. (**e**) The output of a fabricated ROSC operating at 714 kHz input (adapted from [60] with permission from the American Chemical Society). (**f**) Transfer characteristics for the photopatterned ZTO-based TFT after final thermal annealing at various temperatures. (**g**) Schematic illustration of the direct UV photoprocessing on a semiconducting layer (adapted from [61] with permission from John Wiley and Sons).

A TFT is a switching device that turns on and off when carriers (electrons or holes) pass through the semiconductor channel produced by the gate electric field. Logic circuits, such as NOR, NAND, and inverters [50,51], can be constructed by combining these TFTs. In addition, TFTs are used widely in display pixel control arrays and various sensor fields. The usage of ZTO as an active layer in TFTs has many advantages for TFT fabrication because of their high on-current and low off-current, compared to conventional amorphous silicon semiconductors. At the same time, the transparent and flexible physical properties provide advantages for the development of transparent and flexible electronic products [52]. On the other hand, many studies to improve their limited electrical properties [53,54,55,56], complex processes, and difficult patterning are still in progress. In 2011, Kim et al. fabricated ZTO TFTs using a solution-process-based ZTO film and investigated the electrical characteristics and bias stress stability of the ZTO TFT, according to the Zn:Sn ratio of the precursor solution [57]. In the operation of the logic circuit, low-bias stress stability breaks the turn-on-turn-off balance between the two semiconductors and, consequently, loses the noise margin. Therefore, the bias stress stability is one of the important indicators of TFT. Films were synthesized with various Zn:Sn precursor ratios to examine the electrical characteristics of ZTO films, depending on the precursor ratios. As a result, when the amount of Sn was increased, the on-current increased significantly, and the subthreshold swing (SS) and carrier mobility of the ZTO TFT were improved. The fabricated TFT with the maximum carrier mobility was 3.99 cm^2^/V∙s, which occurred in the 1:1 ratio precursor solution. On the other hand, the ZTO film with an extremely high Sn concentration (1:13) had no depletion region and became a conducting film without an off state. Furthermore, the Zn:Sn ratio in the film affects the stability of the device under bias stress. The threshold voltage (VTH) of the device was affected by bias stress. As a result, the VTH of ZTO TFT had the highest ΔVTH at a Zn:Sn ratio of 5:9, with gate bias of 20 V and drain bias of 0 V. Therefore, the 5:9 ratio ZTO film was most sensitive to bias stress. In 2013, Lim et al. enhanced the field-effect mobility and electrical stability of ZTO TFT by doping a ZTO film with alkali metals, such as Li and Na [58,59]. As a result of UV-visible spectroscopy analysis of the film, the bandgap expanded, according to Li doping, which was attributed to the increase in electron carrier concentration. The maximum carrier concentration was observed at 2 mol % Li doping, and the electrical performance of the ZTO TFT was most improved (Figure 2a). Exceeding the optimal Li concentration reduced both the optical bandgap and carrier concentration. In 2014, Kim et al. used an inkjet-printing approach to produce a solution-based N-type ZTO and p-type single-walled carbon nanotubes (SWCNTs) thin film on a substrate, with a photolithography-patterned gate electrode and a ZrO_2_ gate insulating layer [60]. As a result, n-type ZTO TFTs and p-type SWCNT TFTs were fabricated. At each TFT, the ZTO TFTs showed a saturation mobility of 4.4 cm^2^/V∙s and an on–off ratio of 2.6 × 10^6^, and the SWCNT TFTs had a saturation mobility of 1.7 cm^2^/V∙s and an on–off ratio of 3.2 × 10^4^. A complementary inverter circuit was fabricated by combining an n-type ZTO TFT and a p-type SWCNT TFT (Figure 2 b,c), and a five-stage ring oscillator (ROSC) was obtained by combining five complementary inverters, as shown in Figure 2d. The implemented ROSC operates at a high frequency of 714 kHz and has the shortest delay time of 140 ns among the reported ROSCs, using printed semiconductors (Figure 2e). The ZTO film has difficulty in etching because of its high chemical resistance. Patterning has been difficult because the additional etchants for patterning a ZTO film might degrade solution-processed oxide semiconductors. In 2018, Sanctis et al. effectively patterned ZTO TFTs, using a direct photopatterning technique with UV light and methoxyiminopropionato ligands [61]. The ligands and ZTO precursors were stable in visible light but decomposed in UV light, allowing selective film patterning (Figure 2g). UV light completely decomposed the ligands, which were washed out with the solvent without residue. Therefore, there was no decrease in the performance or transparency of the film due to residual substances. As a result, the fabricated ZTO TFT exhibited a high performance of 7.8 cm^2^/V∙s, and the ZTO film showed high optical transmittance of more than 90%, as shown in Figure 2f. In 2019, Wang et al. fabricated the ZTO TFTs with a performance comparable to a-IGZO TFTs, using self-aligned top-gate (SATG) technology [62]. A N_2_O plasma treatment, photolithography, and Ar plasma treatment was performed to adjust and improve the electrical characteristics of the a-ZTO film deposited via sputtering. The overlap between the source/drain and the gate electrodes of the ZTO TFT was minimized to reduce the parasitic capacitance [63]. Furthermore, with the N_2_O plasma treatment, the ZTO channel layer achieved a high carrier mobility of 12.1 cm^2^/V∙s and a low SS of 0.3 V/dec. The ZTO conductive layer treated with Ar plasma has a low resistance between the film and electrodes. The performance improvement of the ZTO film with the N_2_O plasma treatment was investigated by XPS. As a result, the oxygen vacancies in the film without the N_2_O plasma treatment and the film after treatment were decreased from 20.9% to 13.2%. This suggests that the N_2_O plasma treatment compensated for the oxygen vacancies and improved the Mo-bond strength of the ZTO layer, resulting in a low interfacial trap density between the a-ZTO and gate insulator.

### 2.3. Flexible Electronics

**Figure 3:**
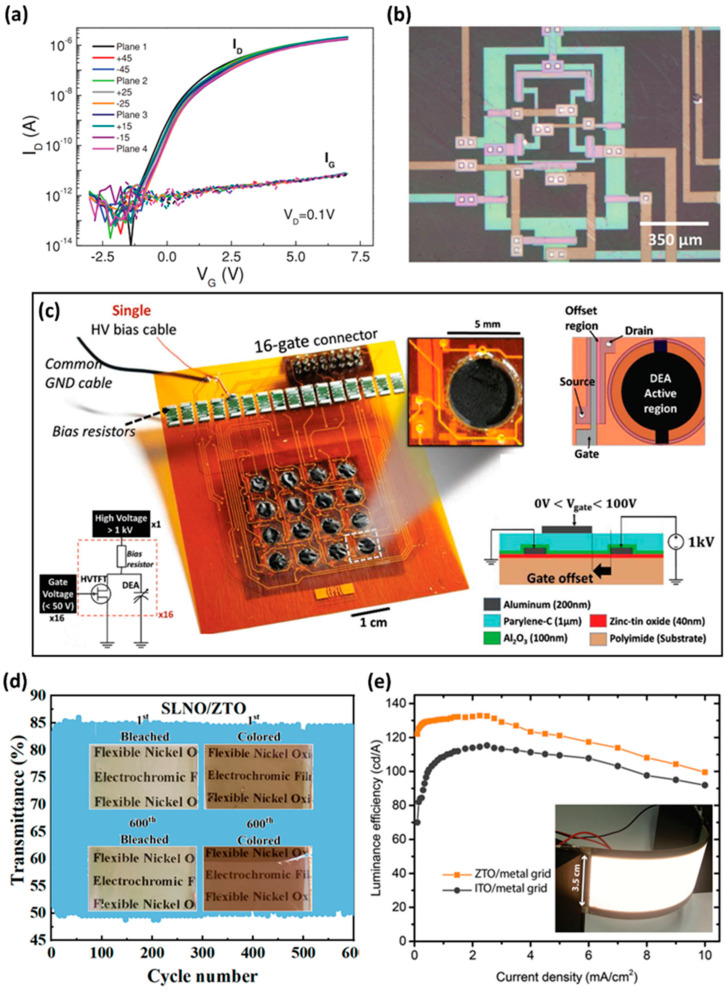
(**a**) Transfer curve in the bending state of ZTO TFT with improved performance using hydrogen incorporation and multilayer/multi-component dielectric and (**b**) image of differential amplifier demonstrated using ZTO TFTs (adapted from [68] with permission from John Wiley and Sons). (**c**) Flexible out-of-plane DEAs and flexible high-voltage thin-film transistors (HVTFTs) array consisting of a matrix of 4 × 4. The DEA is controlled by HVTFTs with a single 1.4 kV supply (adapted from [70] with permission from John Wiley and Sons). (**d**) The 500 nm light transmittance change in the ZTO buffer layer EC film operated in an electrolyte during 600 cycle tests (adapted from [71] with permission from the American Chemical Society). (**e**) Luminance efficiency versus current density in fabricated flexible sm-OLEDs (adapted from [16] with permission from John Wiley and Sons).

Recently, TFTs on flexible substrates have emerged as a critical element in flexible applications, such as flexible displays, electronic skin, and smart textiles [64]. Oxide semiconductors, such as ZTO, have outstanding characteristics, such as strong electrical performance and good flexibility. In particular, in the case of ZTO, it offers excellent visible light transmittance, with good electrical performance. Therefore, ZTO can be used in a variety of flexible electronic applications, including flexible and transparent displays in the future. The TFTs fabricated using common solution techniques have poor performance because of the unexpected impurities, including oxygen, water molecules, and organic residues in the residual precursors. To address this issue, additional high-temperature annealing at 500 °C was used to fabricate a high-performance ZTO film with increased crystallinity. However, as is well known, flexible substrates are made of polymer-based films, such as polyimide (PI), polyethylene terephthalate (PET), polyethersulfon (PES), and polydimethylsiloxane (PDMS), which are vulnerable to high temperatures of 300 °C or higher. Consequently, to apply the ZTO into the flexible applications, the following approaches can be considered: (1) photo-annealing using UV light rather than high-temperature annealing, (2) combustion chemistry method, and (3) transferring the ZTO film after synthesizing on a rigid substrate with high thermal stability [25,65,66]. In 2016, Ha et al. investigated a simple and reproducible method to implement a high-performance, transparent and flexible ZTO TFT with low-temperature microwave annealing (~100 °C) and a ferroelectric poly (vinylidenefluoride-co- trifluoroethylene) (PVDF-TrFE) copolymer film [67]. The density of the trap state was lowered by the charge screening of the interface, owing to the dipole interaction caused by the encapsulation of the ferroelectric copolymer film layer formed on the ZTO film. This improved the performance of the ZTO film. As a result, compared to the TFT without encapsulation, V_TH_ shifted to 0 V; the off-state current reduced approximately 20-fold, and SS decreased from 1.42 V/dec to 0.4 V/dec. The TFT showed highly reliable operation, even after 5 × 10^4^ seconds of negative bias illumination stress (NBIS). The ZTO TFT, fabricated as a sandwich structure between two high-k dielectric films constructed of ZrO_2_ and PVDF-TrFE, showed increased device performance and stability, while also achieving low-process-temperature characteristics, suitable for flexible electronics. In 2018, Fernandes et al. fabricated amorphous-ZTO TFTs on polyethylene naphthalene (PEN) flexible polymer substrates in a low-temperature process of less than 180 °C [68]. Several aspects have been investigated to achieve performance levels equivalent to IGZO devices at these low processing temperatures, including hydrogen incorporation during ZTO sputtering and integration with a high-k multilayer/multi-component dielectric. As a result, stable performance in the bending state, a carrier mobility of approximately 5 cm^2^/V∙s, and SS of 0.26 V/dec were obtained, as shown in Figure 3a. Based on this, they implemented a flexible inverter [69] and differential amplifier with a gain of 17 dB and a unity gain frequency of 40 kHz (Figure 3b) [68]. Therefore, hydrogen incorporation during ZTO deposition was effective in improving the electrical performance of the device, and the applied high-k/multi-component insulating film was effective in protecting the performance from mechanical deformation. In 2017, Marette et al. fabricated a solution-based ZTO high-voltage thin-film transistor (HVTFT) on a flexible PI substrate that was structurally (at channel length, offset gate) and materially (double-insulating film) optimized and used a high voltage of 1 kV to drive the compliant dielectric elastomer actuators (DEA), fabricated on a flexible substrate [70]. When a voltage of 1 kV is applied to soft actuator DEA, charges accumulate on the DEA electrodes and then actuates, deflecting out of plane. The activated DEA automatically returns to its initial position when electrical energy is not supplied. The circuit structure is an N-type inverter composed of a HVTFT, a pull-up resistor, and the DEA at the output node. When ZTO HVTFT is turned on by a gate voltage of 50 V, a high voltage of 1 kV flows from the source and drains to the ground. Conversely, when the HVTFT is turned off, a 1 kV voltage is supplied to the DEA, which operates the DEA. To reduce the influence of a high electric field on the HVTFTs, they used a channel, 500 µm in length, for a 5 mm width (W/L = 10). High-field effects showing a similar short-channel effect can be reduced by increasing the channel length. In addition, a double-insulating layer consisting of Al_2_O_3_ and perylene-C, and an offset gate structure, were used to enhance the operating voltage. Owing to these multi-faceted efforts, the measured breakdown voltage of ZTO HVTFT reached 1.1 kV, which is the highest value among reported TFTs. The fabricated ZTO HVTFT and DEA-array-integrated device works properly at a radius curvature of 2.5 mm. This demonstration of low-voltage control of a matrix of kV scale actuators opens the way for the DEAs to be used in soft robotics, haptic displays, and flexible braille displays, among other applications. Furthermore, because the ZTO HVTFT can operate with a high voltage, ZTO HVTFT can be used in various high-voltage circuit applications (Figure 3c). In 2021, Lou et al. used ZTO films as a buffer layer to improve the cycling stability of an existing NiO_x_-based flexible electrochromic (EC) film [71]. Existing NiO_x_-based EC films have poor stability, in that the transmittance decreases gradually with an increasing number of operating cycles. This phenomenon was caused by the collapse of the internal nanocolumnar structure due to the harsh injection and extraction of Li ions. This problem can be solved by inserting a ZTO buffer layer to prevent the collapse of nanocolumnar and improve the rate of Li ion transport. As a result, as shown in Figure 3d, the fabricated EC film with the added ZTO buffer layer has a stable EC operation, even after 600 on–off cycles and high mechanical stability in 2000 bending tests. This EC and mechanical stability have promise in future high-performance EC devices in commercial applications. In 2016, Morales-Masis et al. developed a transparent flexible electrode to replace the ITO in OLED manufacturing by optimizing the tin ratio in the ZTO film [16]. The fabricated ZTO flexible electrode was synthesized at a low temperature of 60 °C and was easily compatible with flexible substrates, had a uniform surface (0.25 nm RMS), high carrier mobility of up to 21 cm^2^/V∙s, and high conductivity of 245 S cm^−1^, with a low visible light spectral absorption of less than 5%. Finally, ZTO flexible transparent electrodes are suitable as anodes for flexible OLED displays because they have similar or better conductivity than conventional ITO transparent electrodes and are free of indium scarcity. Figure 3e shows an OLED that operates with a flexible transparent electrode.

### 2.4. Memory Applications

**Figure 4:**
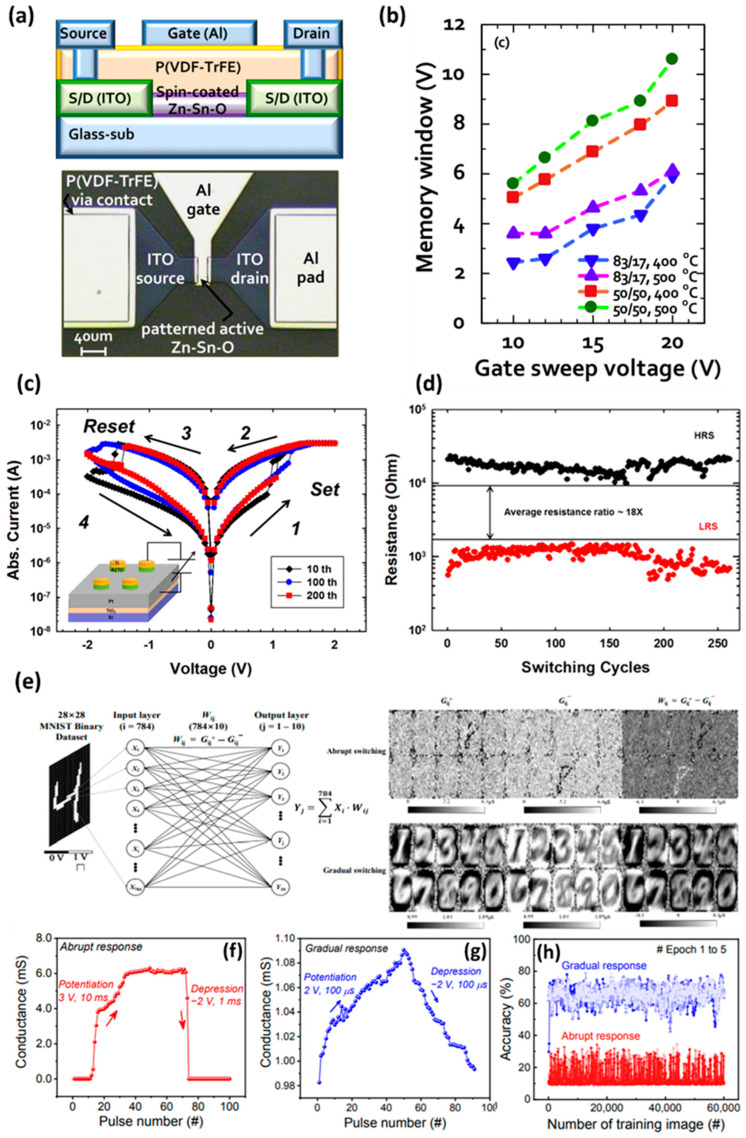
(**a**) Schematic diagram and optical image of a top-gate MTFT fabricated from the solution-processed ZTO channel and the P(VDF-TrFE) gate insulator. (**b**) Variation of memory window according to the annealing and composition ratio of zinc and tin in the ZTO film. The sweep range of V_G_ was performed from ±10 to ±20 V (adapted from [74] with permission from Elsevier). (**c**) The bipolar resistance switching I-V curves in the fabricated Ti/ZTO/Pt structure device. (**d**) Switching cycling test with a current compliance of 3 mA (adapted from [75] with permission from AIP Publishing). (**e**) Device designed in the system simulation framework for MNIST pattern categorization: single-layer neural network for MNIST pattern categorization. A synaptic weight map showing the results after learning 60,000 training images using abrupt and gradual switching experimental models. Potentiation and depression results according to the (**f**) abrupt response and (**g**) gradual response of the fabricated memristor devices. (**h**) Accuracy of pattern recognition according to gradual response and abrupt response (adapted from [76] with permission from Elsevier).

Recently, there have been several attempts to apply oxide semiconductors, including ZTO, to the memory field using the advantages of high performance and the simple process. ZTO-based memory shows stable memory operation characteristics based on high charge mobility and on–off ratio [72]. In addition, ZTO offers high transparency and mechanical flexibility, making it a promising candidate for transparent or flexible memory devices. Oxide-semiconductor-based memory is an ideal candidate for future transparent or flexible devices because oxide semiconductors offer more transparency and mechanical flexibility than typical silicon semiconductors. In 2016, Li et al. implemented ZTO TFT-based nonvolatile charge trapping memories (CTMs) using nickel nanocrystals as charge trapping regions [73]. In the fabricated optical memory, the V_TH_ shifted up to 7 V with a positive gate programming voltage of 40 V, but the programmed state was not erased at a negative gate voltage. On the other hand, the programmed optical memories can be erased easily by irradiating visible light for one second with a gate voltage of −10 V. ZTO TFTs without the trapping layer were compared to verify the nickel-nanocrystal-trapping layer. There was no change in V_TH_, even at a gate voltage of 50 V, which is higher than the existing programming voltage. This suggests that electrons injected in the ZTO channel can be effectively trapped and detrapped from the Ni NCs charge-trapping layer. In addition, in memory retention, even after 10^4^ seconds, the initial on–off ratio of 5 × 10^5^ was maintained at 4 × 10^4^. Hence, the fabricated CTM is suitable for saving data in the long term. In 2010, Yoon et al. used a solution-processed ZTO film with a poly(vinylidene fluoride-trifluoroethylene) ferroelectric gate insulating layer to develop a nonvolatile memory thin-film transistor (MTFT) [74]. The MTFT properties were compared and optimized based on the zinc and tin composition ratio and annealing temperature of the ZTO semiconductor film. As a result of optimization, the ZTO film showed the highest performance under a zinc:tin ratio of 50:50 and an annealing temperature of 500 °C. The carrier mobility, SS, and on–off ratio were approximately 15.8 cm^2^/V∙s, 1.1 V/dec, and 6.4 × 10^7^, respectively. As shown in Figure 4a, the MTFT fabricated using the optimized ZTO film was a top gate TFT structure with a ferroelectric film functioning as a gate insulating film. The magnetic field inside the film was aligned with the electromagnetic field formed by the programming gate bias and the erasing gate bias, resulting in a ferroelectric-based memory device that shifts the V_TH_ of the TFT. The memory window showed a V_TH_ shift of 8.1 V from a gate bias of ±15 V in the optimized ZTO film (Figure 4b). As a result, interesting memory structures based on ferroelectric magnetic field alignment can be synthesized using a simple solution technique, potentially improving the functionality of next-generation electronic devices and systems. In 2012, Fan et al. fabricated resistive random-access memory (RRAM) using a sputtered Al-doped ZTO film as a resistive switching layer [75]. RRAM repeats the formation and removal of conductive channels inside the semiconductor material between the electrodes to control the low-resistance (LRS) and high-resistance (HRS) memory states. The fabricated ZTO-based RRAM operates by forming and removing a conductive filament containing oxygen vacancies and then repeating the set and reset processes at approximately 1.1 V and −1.6 V, respectively (Figure 4c). Through a cycle test with 256 cycles, these iterations of setting and resetting were confirmed to be reliable. In addition, as shown in Figure 4d, the resistance between the LRS and HRS differed by an average of 18 times at a read voltage of 0.2 V, and the LRS and HRS, once set or reset, were maintained stably for 10^4^ seconds, even under a bias stress of 200 mV. In 2021, Ryu et al. used a sputtering procedure to produce a transparent resistive memory (memristor) with an ITO/ZTO/ITO structure [76,77]. For applications in neuromorphic systems [77,78], the electrical characteristics of the memristors corresponded to the biological synapse properties. The gradual transient pulse response to the pulse input obtained from the memristor is suitable for hardware-based neuromorphic systems. Therefore, pattern recognition simulation using a single-layer neural network was performed, as shown in Figure 4e, to confirm the neuromorphic potential. In a wide voltage sweep range, the fabricated resistive memory showed an abrupt switching operation, but gradual switching operation was achieved when the sweep range was limited to 3 V. In the conductance change according to the pulse shown in Figure 4f,g, potentiation and depression appear rapidly and irregularly in the abrupt switching operation. On the other hand, the gradual switching operation in the limited voltage sweep generated constant and regular potentiation and depression, as shown in Figure 4g. Because of these results, the pattern recognition simulation accuracy for these two switching behaviors was compared (Figure 4h), and the memristor with gradual switching showed high accuracy. This transparent neuromorphic memristor device might be considered a promising element for future artificial synaptic implementations based on these results.

### 2.5. Solar Cell Applications

**Figure 5:**
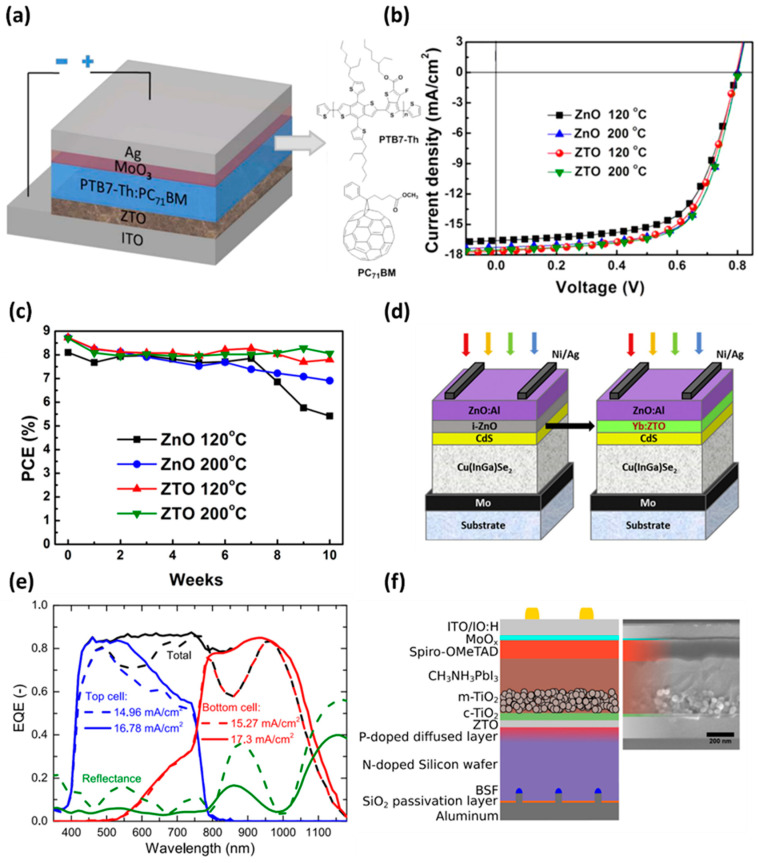
(**a**) Schematic image of ZTO-based device and molecular structures of PTB7-Th and PC_71_BM active layer. (**b**) The J-V characteristics of ZnO and ZTO ETL-based devices depend on the heat treatment temperature. (**c**) Long-term operational stability of fabricated various ETL-based devices (adapted from [79] with permission from the American Chemical Society). (**d**) Schematic illustration of a Cu(InGa)Se_2_-based solar cell using Yb:ZTO as a potential down-converter layer (adapted from [81] with permission from Elsevier). (**e**) J-V characteristics comparing the external quantum efficiency (EQE) of each upper cell and lower cell of the mesoscopic perovskite/silicon homojunction and the total EQE after monolithic integration. (**f**) Illustrated and cross-sectional SEM images of mesoscopic perovskite/silicon homojunction monolithic tandem solar cells integrated using a ZTO buffer layer (adapted from [83] with permission from AIP Publishing).

In the solar cell field, ZTO is attracting attention as a suitable replacement for ITO and IZO, owing to low light absorption and good conductivity because of its large bandgap. In the case of perovskite, a conductive film with strong heat resistance was required because of its high-temperature heat-treatment process. The ZTO maintains its physical properties without forming thermal degradation at high temperatures (~500 °C), having high thermal stability. Therefore, it is recommended as an excellent buffer layer in perovskite solar cell applications. In 2017, Wei et al. fabricated an electron transport layer (ETL) of organic solar cells (OSC) using a ZTO thin film [79]. Figure 5a presents the construction of the fabricated OSC device, and the device was stacked in the sequence ITO/ZTO/ PTB7-Th:PC71BM/MoO_3_/Ag. The ZTO electron transport layer has a high-power conversion efficiency (PCE) of 9.32%, which is improved compared to the existing ZnO layers. In particular, as shown in Figure 5b, ZnO and ZTO fabricated at a low temperature (120 °C) have a considerable PCE difference of 8.46% and 9.02%, respectively. In addition, the PCE of the ZnO device decreased rapidly after seven weeks, but the PCE of the ZTO device remained stable at 9% or higher until the 10th week (Figure 5c). In 2016, Pimachev et al. improved the quantum efficiency by 150%, using the ZTO charge transport layer and Mn-doped CdSe quantum-dot-sensitized solar cells [80]. A typical CIGS solar cell requires a good electrical and optical buffer layer between the CIGS and the bottom semiconductor layer. A high bandgap and simplicity of bandgap adjustment are crucial considerations in the solar cell buffer layer to achieve proper electrical properties and prevent unnecessary light absorption. The low bandgap of CdS, which is commonly used as a buffer layer, causes parasitic absorption in low-wavelength lights. At the same time, it has issues, such as the non-vacuum process being incompatible with the CIGS process and high cadmium toxicity. Consequently, ZTO is presented as an effective buffer layer to replace CdS in solar cells. The bandgap can be controlled efficiently, and operate stably, even at high temperatures, and exhibit high light transmittance by controlling the zinc and tin ratio. In addition, the non-toxicity of zinc and tin is also an advantage. In 2020, Park et al. developed a ZTO potential down-converter layer that combines the optical transparency of ZnO with the high electrical conductivity of SnO_2_ to replace the existing ZnO conductive layer used in solar cells (Figure 5d) [81]. In addition, these ZTO films were doped with Yb, a rare earth element, to improve their electrical performance. The fabricated Yb:ZTO film was compatible with Cu(InGa)Se_2_ photovoltaic cells film [82], and it exhibited high-electrical-current density and cell efficiency, suitable for solar cell applications. Furthermore, the Yb:ZTO film showed higher energy transfer efficiency at an optimized Sn sputtering power of 15 W and a substrate temperature of 100 °C compared to other conditions. This is a good example of improving the solar cell efficiency using rare earth elements. The Yb doping technique and ZTO film can be considered attractive candidates in the field of solar cells. In 2016, Werner improved the quantum efficiency of solar cells by monolithically integrating silicon cells with perovskite materials that absorb different wavelengths (Figure 5e) [83]. The integrated buffer layer of the two solar cell layers should operate stably, even at the high crystallization temperature of perovskite, and required high transmittance of light at wavelengths between 600 and 1200 nm. Although traditional ITO and IZO transparent electrodes have poor high-temperature stability, ZTO has a high light transmittance and is stable at high temperatures, making it an ideal candidate for monolithic integrated layers, as shown in Figure 5f.

### 2.6. Sensors

**Figure 6:**
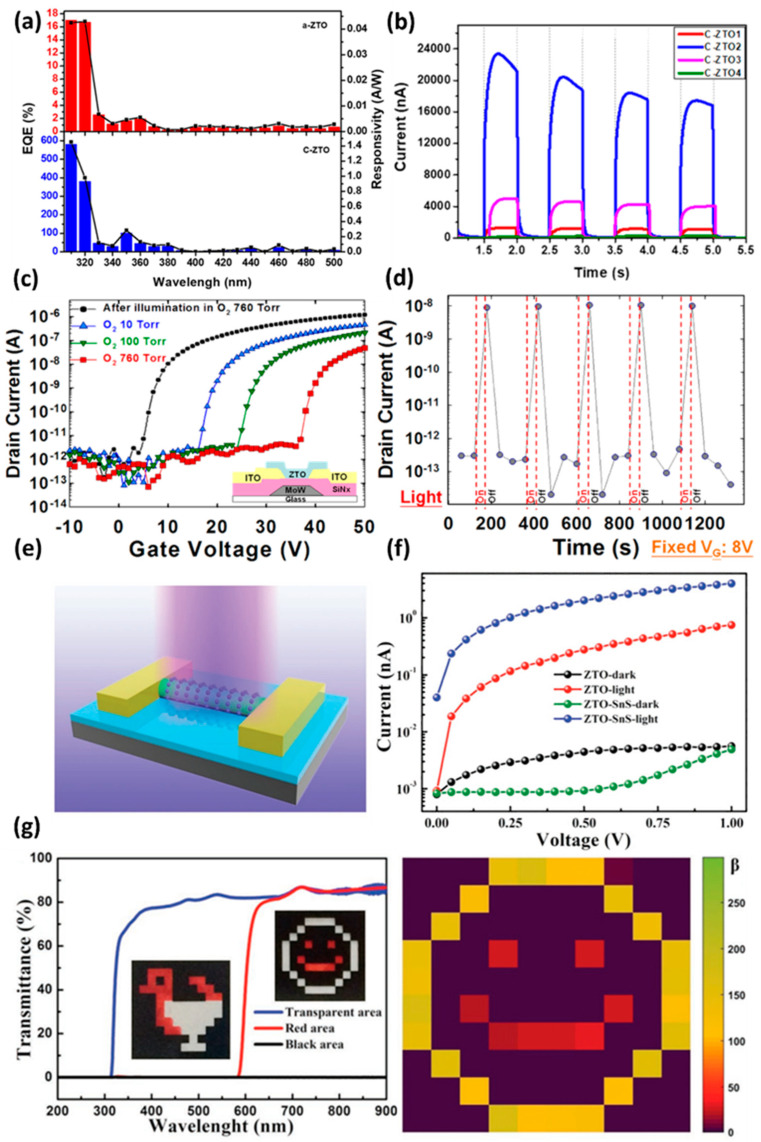
(**a**) Photon-to-electron conversion efficiency according to the crystallinity of the fabricated a-ZTO-MS and c-ZTO-MS and the wavelength of the irradiated light. (**b**) I-V characteristics of photocurrent difference of c-ZTO-MS monolayers with varied MS diameters at a UV wavelength of 254 nm. The MS has diameters of 1.51 μm, 1.24 μm, 0.99 μm, and 0.79 μm, respectively (adapted from [87] with permission from American Chemical Society). (**c**) Transfer characteristics of the fabricated ZTO TFTs in different oxygen partial pressures (10 torr, 100 torr, and 760 torr) and after illumination of visible light, respectively. (**d**) Repeated oxygen adsorption/desorption cycle depending on light irradiation, the I_DS_ decreases upon the adsorption of oxygen and increases upon the desorption of oxygen by visible light (the fixed gate voltage of 8 V and drain voltage at 1 V) (adapted from [97] with permission from AIP Publishing). (**e**) Schematic diagram of ZTO NW and a SnS QDs decorated photodetector fabricated on SiO_2_/Si substrate. (**f**) I-V curve of photodetectors made of a pristine ZTO NW and ZTO NW decorated with SnS QDs under 300 nm UV illumination. (**g**) The flexible ZTO NW-based 10 × 10 image sensor array fabricated on a PET substrate and decorated with SnS QDs can detect bicolors, as shown in the image below (adapted from [98] with permission from John Wiley and Sons).

Oxide semiconductors can be produced with various structures, such as thin films, nanoparticles [84,85,86], microspheres [87], nanomesh [88], and nanowires [84,89], and have appropriate photoreactivity. Therefore, they are applied in various fields, such as gas sensors [90] and light sensors [91,92,93,94]. In particular, The ZTO is actively used for UV sensors that require a wavelength in a range of 200 to 400 nm and energy distribution range of 3.1 to 6.2 eV. Due to the wide bandgap of the ZTO, as large as ~3.6 eV [95], the ZTO offers a desirable UV absorption layer. Furthermore, the energy bandgap can be manipulated depending on the Sn ratio in the ZTO [96], which provides a tunability of appropriate bandgap according to the sensing target wavelength. In 2020, Jung et al. developed a highly efficient UV photodetector using ZTO microspheres (MS). The photodetection performance was compared according to the crystallinity of ZTO, and the performance was optimized according to the diameter of the ZTO-MS [87]. The external quantum efficiency for UV improved as the crystallinity of ZTO was increased. Amorphous ZnSnO_3_ (a-ZTO) and crystalline Zn_2_SnO_4_ (c-ZTO) showed a larger difference in efficiency of 16.7% and 580.9%, under UV light of 310 to 320 nm, respectively (Figure 6a). The device with the highest quantum efficiency among the c-ZTO-based detectors had an MS diameter of 1.24 μm, which was a 51-fold improvement over the device with the lowest performance. This mechanism was investigated by XPS. The ZTO-MS with many oxygen vacancies and a low hydroxide content showed higher device performance. 

The UV photodetector, fabricated based on the optimized result, successfully repeated the switching operation by irradiating a light pulse with a frequency of 0.5 s, as shown in Figure 6b. In 2012, Chen et al. used a zinc–tin-oxide-based TFT produced by a sol–gel technique to develop an oxygen sensor with remarkable stability and performance [97]. The developed oxygen sensor was controlled by the oxygen adsorption and desorption in the ZTO active layer by irradiated light. In the dark state, the ZTO active layer increased the depletion region by adsorbing oxygen ions from the environment. In the ZTO TFT transfer curve, V_TH_ shifted to the positive side as the oxygen concentration increased, as shown in Figure 6c. V_TH_ shifted rapidly to the negative direction when the oxygen-adsorbed ZTO layer was irradiated with visible light for 120 s. This behavior was caused by the electron–hole pair (EHP) generated inside the ZTO active layer by visible light. The hole from the EHP was released by interacting with the adsorbed oxygen ion, and the electron was transferred via the ZTO active layer. As a result, in the fabricated oxygen sensor, the oxygen desorption state (on-state) and adsorption state (off-state) were identified with a high on–off ratio of 10^4^. Moreover, it operated stably in repeated cycles (Figure 6d). In 2017, Li et al. developed a flexible image sensor decorated with SnS QDs, based on ZTO nanowires (NW) (Figure 6e) [98]. Compared to the pristine ZTO NW image sensor, the SnS QD-decorated image sensor device showed a much higher 1.61 × 10^6^ photoconductive gain. This was caused by electrons from the EHP generated by the SnS QD layer being injected into the ZTO and forming a high photocurrent, through efficient bandgap matching (Figure 6f). In addition, an image sensor with a 10 × 10 array was produced and irradiated with two colors, white light and red light, as shown in Figure 6g. The mapping image showed that each had a different amount of current (β = I_light_ / I_dark_), depending on the light source, and effectively distinguished the two types of light. The outstanding mechanical durability and flexibility of the ZTO-SnS-based photodetector over 5000 cycles and long-term stability for 63 days highlight its potential for future flexible device applications as flexible image sensors.

## 3. Conclusions

This review provided an overview of the recent advances in ZTO-based electronic and energy applications and the synthesis of ZTO films. ZTO films are promising materials because of their Earth abundance and high process compatibility. Furthermore, the semiconducting properties of ZTO films, with high transparency and flexibility, allow their extensive use in various device applications, including thin-film transistors, memory devices, solar cells, and sensors. On the other hand, there are still challenges to overcome for practical applications and systems using the ZTO films:

(1) A more elaborate ZTO-film-patterning process is needed to improve the scalability of ZTO-based devices. A robust and stable patterning process based on ZTO should be accompanied because scalable patterning technology is crucial for the success of silicon-based electronics.
(2) Research efforts on the device process yield and reproducibility for commercialization are still insufficient. For more practical use of ZTO films, an analysis of single-device characteristics, yield verification, and reproducibility evaluations through multiple device tests are needed.
(3) For the ZTO-based thin-film transistor aspect, development is required at the level of a more complex circuit beyond a single device or inverter circuit. The research focused only on improving the charge mobility of ZTO unit transistors. On the other hand, interest in complementary circuit integration and the development of p-type transistors compatible with the ZTO film is increasing.

However, despite these challenges, ZTO-based applications are expected to expand in the following directions. Due to the extremely low off-state current, the ZTO can be applied to more complex ICs, such as microprocessors [99] or RFID transponder circuits [100]. Furthermore, by forming a hybrid material combination of ZTO and other materials, multi-valued circuits [101,102] or charge transport enhancement can be attained [103]. As another application, the high conductivity and thermal stability of ZTO provide a desirable conductive layer in solid-state sodium batteries [104]. In summary, ZTO is still a promising key material for electronic and energy devices. If there is continuous effort and interest in the development of ZTO-based devices, ZTO will become a valuable material for eco-friendly next-generation electronics.
